# The increasing prevalence of myopia in junior high school students in the Haidian District of Beijing, China: a 10-year population-based survey

**DOI:** 10.1186/s12886-017-0483-6

**Published:** 2017-06-12

**Authors:** Yan Li, Jia Liu, Pengcheng Qi

**Affiliations:** 1 Department of Ophthalmology, College of Optometry, Peking University Health Science Center; Center of Optometry, Peking University People’s Hospital; Beijing Key Laboratory of Diagnosis and Therapy of Retinal and Choroid Diseases, Beijing, 100044 China; 2 Key Laboratory of Vision Loss and Restoration, Ministry of Education, Department of Ophthalmology, Peking University People’s Hospital, Xizhimen South Street 11, Xi Cheng District, Beijing, 100044 China

**Keywords:** Myopia, Prevalence, Junior high school, Haidian District, Beijing

## Abstract

**Background:**

Myopia is a leading cause of preventable blindness. Although, multiple cross-sectional epidemiological studies have confirmed that there is a high prevalence of myopia in high school-aged students in China. However, few longitudinal studies have been performed to assess junior high school students. In the present study, we investigate changes in the prevalence of myopia in third year junior high school (grade 9) students in the Haidian District of Beijing, China, from 2006 to 2015.

**Methods:**

A retrospective, longitudinal cohort study was performed over 10 years. A total of 37,424 third-year middle school (grade 9) students from 8 junior high schools in Haidian district, Beijing, were included. Participants underwent a comprehensive ophthalmic examination in which they were evaluated using autorefraction under cycloplegia and submitted to retinoscopy to assess accuracy. According to the spherical equivalent refraction (SER) of the right eye, subjects were separated into the following groups: non-myopia, −0.5 ≤ SER diopters (D); low myopia, −3.0 ≤ SER < −0.5 D; moderate myopia, −6.0 ≤ SER < −3.0 D; and high myopia, SER > −6.0 D. The following characteristics were measured: refractive error; the proportion of subjects with non- myopia, low myopia, moderate myopia and high myopia; and the difference in the prevalence of myopia between male and female subjects.

**Results:**

From 2006 to 2015, the prevalence of non-myopia (from 44.05% to 34.52%) and low myopia (from 32.27% to 20.73%) decreased, while the prevalence of moderate myopia (from 19.72% to 38.06%) and high myopia (from 3.96% to 6.69%) significantly increased. For refractive error, the worse eye was −2.23 ± 2.42 D (median, −1.75; range − 12.75 to +8.50) in 2006 and −3.13 ± 2.66 D (median, −2.75; range − 12.75 to +8.50) in 2015. When the entire population was considered, the overall prevalence of myopia increased from 55.95% in 2005 to 65.48% in 2015. There was a significant positive relationship between the year and the prevalence of myopia in both girls and boys. Girls were more likely than boys to have myopia (odds ratio, 1.43 [95% CI, 1.14–1.96]), especially moderate myopia, and the prevalence of moderate and high myopia were higher in girls than in boys.

**Conclusions:**

During the last 10 years, the prevalence of myopia significantly increased on an annual basis among third-year junior high school students in the Haidian District of Beijing, China. The total prevalence of myopia was significantly higher in girl than in boy participants. The refractive status of this age group deserves particular attention.

**Electronic supplementary material:**

The online version of this article (doi:10.1186/s12886-017-0483-6) contains supplementary material, which is available to authorized users.

## Background

Myopia is a leading worldwide cause of preventable blindness, especially in children and young adults [1, 2]. Recent epidemiological studies have revealed that the prevalence of myopia is rapidly increasing globally, especially in East and Southeast Asian countries. For example, in Taiwan, the prevalence of myopia among 16- to 18-year-old children increased from 74% in 1983 to 84% in 2000 [3]. In Japan, the prevalence among 7-year-olds increased from 39% in 1984 to 59% in 1996 [4]. Additionally, a series of surveys demonstrated that the prevalence among teenagers is approximately 96.5% in Korea [5, 6], 81.6% in Singapore [7], and 95.5% in Shanghai, China [8].

In China, the school education system includes pre-school, primary school, junior high school, and senior high school. Since 1986, China’s governmental Ministry of Education has implemented 9 years of compulsory education, including 6 years of primary school (grades 1 to 6) education and 3 years of junior high school (grades 7 to 9) education [9]. At the end of the third year of junior high school, most of the students continue to senior high school (grades 10 to 12), which is followed by entering a university, especially in urban cities. The remaining students who do not go to senior high school go to work, usually in rural areas. Because a high level senior high school is associated with a higher likelihood of entering a distinguished university and because attending such a university is viewed as an indicator of access to better work opportunities, grade 9 and grade 12 students are under a particularly high amount of study pressure. Previously, multiple cross-sectional epidemiological studies have confirmed that there is a high prevalence of myopia in high school-aged students in China [10–12]. Additionally, several studies have focused on the incidence of myopia in grade 12 students and shown that the prevalence of myopia is estimated to be approximately 80% in 17-year-olds [9]. However, relatively few longitudinal studies have been performed to assess junior high school (grade 9) students, and large-scale retrospective studies are the most accurately methods to evaluate changes over time [4].

In the present study, we analyzed longitudinal data obtained from students of the Haidian District of Beijing, China from 2006 to 2015 to track changes in the prevalence of myopia in third-year junior middle-school (grade 9) and to evaluate current trends with the aim of providing guidance for the future management of myopia in China.

## Methods

### Study population and sampling

The present retrospective, longitudinal cohort study was conducted in the Haidian District of Beijing, China from September 2006 to September 2015. Out of 73 junior high schools, 8 were randomly selected. All third-year students in the selected schools were invited to participate in the study, and the refractive status of all of the evaluated students were routinely collected each year. The survey was conducted by a research group that consisted of two qualified ophthalmologists and two optometrists. All participants were registered, including their name, sex, age, and the name of their school.

### Ethics, consent and permissions

Ethical approval was obtained from the Ethics Review Board and the Ethics Committee of Peking University People’s Hospital. Informed written consent as obtained from at least one parent or guardian of each child. The study adhered to the guidelines of the Declaration of Helsinki.

### Eye examination and VA measurements

The ophthalmologic examination consisted of measurements of uncorrected visual acuity (VA) and best-corrected visual acuity (BCVA), a slit lamp-based examination of the anterior ocular segment by an ophthalmologist, assessments of tonometry (noncontact tonometer; Canon TX-F Full-Auto Tonometer; Canon Co., Tokyo, Japan), ocular motility, and binocularity, and an evaluation to determine the presence of strabismus. Cycloplegia was achieved by instilling at least five drops of 1% cyclopentolate (Alcon, Fort Worth, TX, USA) in intervals of 5 min before obtaining autorefraction measurements (ARK-900; Nidek, Tokyo, Japan). During the refractometry, each eye was measured at least three times [5].

VA was performed at 5 m in examination rooms that were illuminated to approximately 500 lx using a Standard Logarithmic Visual Acuity E chart (Wenzhou Xingkang Medical Technology Co., Ltd., Zhejiang, China). The BCVA was recorded as the smallest size at which at least three optotypes were correctly identified. BCVA was measured using subjective refraction by two senior experienced optometrists.

The spherical equivalent refraction (SER) was calculated as the spherical value of the refractive error plus half of the cylindrical value. Using the SER for the worst eye of each subject, the refractive error (RE) was defined as non-myopia (−0.5 ≤ SER diopters (D), including hyperopia and emmetropia), low myopia (−3.0 ≤ SER < − 0.5 D), moderate myopia (−6.0 ≤ SER < −3.0 D), or high myopia (SER > −6.0 D).

### Statistical analysis

The data were analyzed using the SPSS software program (SPSS, Chicago, IL, USA) and GraphPad Prism software, version 5.0 (GraphPad Software Inc., San Diego, CA, USA). In this report, all data were analyzed using the worse eye of each participant. Median [interquartile range (IQR)] and percentage values were reported in the descriptive analyses for continuous and categorical variables, respectively. Chi-squared tests were used to compare differences in myopia between boy and girls. Odds ratios (ORs) and their 95% confidence intervals (CI) are presented. All *P* values were obtained using *t* tests, and a value less than 0.05 was considered statistically significant.

## Results

### Characteristics of the study population

Over 10 years, 37,424 eligible students were invited to participate (Additional file 1). The students had a mean age of 15.25 ± 0.46 years old (median: 15.6 years old; range, 14–16 years old), and 17,925 of the students were boys (47.90%), while 19,499 were girls (52.10%). Table 1 shows the demographic characteristics of the study participants.

**Table 1:** Demographic characteristics of the participants in the Haidian District of Beijing, China

Year	*N* ^a^	Gender (Boys/Girls) (%)
2006	3657	1742/1915 (47.63/52.37)
2007	3615	1720/1895 (47.58/52.42)
2008	3662	1728/1934 (47.19/52.81)
2009	3697	1833/1864 (49.58/50.42)
2010	3897	1853/2044 (47.55/52.45)
2011	3784	1926/1858 (50.90/49.10)
2012	3816	1752/2064 (45.91/54.09)
2013	3787	1816/1971 (47.95/52.05)
2014	3833	1783/2050 (46.52/53.48)
2015	3676	1772/1904 (48.20 /51.80)
Total	37,424	17,925/19499 (47.90/52.10)

^a^ *N* = number

### Change in refractive error

The mean spherical equivalent refractive error (SERE) significantly increased during the 10-year study period from −1.92 ± 2.26 D [media −1.50, range − 12.75 to +8.50] in right eyes, −2.03 ± 2.32 D [media −1.50, range − 11.25 to +7.25] in left eyes and −2.23 ± 2.42 D [media −1.75, range − 12.75 to +8.50] in worse eyes in 2006 to −2.75 ± 2.51 D [media −2.50, range − 12.75 to +8.25] in right eyes, −2.95 ± 2.61 D [media −2.75, range − 13.00 to +10.75] in left eyes and −3.13 ± 2.66 D [media −2.75, range − 12.75 to +8.50] in worse eyes in 2015 (Table 2). The obtained refractive errors were not normally distributed (*P* < 0.001).

**Table 2:** Refractive error (diopters; spherical equivalent) in participants in the Haidian District of Beijing, China

	Right eye				Left eye				Worse eye			
Year	Mean	Median	SD^a^	Min^b^, Max^c^	Mean	Median	SD^a^	Min^b^, Max^c^	Mean	Median	SD^a^	Min^b^, Max^c^
2006	−1.92	−1.50	2.26	−12.75, +8.50	−2.03	−1.50	2.32	−11.25, +7.25	−2.23	−1.75	2.42	−12.75, +8.50
2007	−1.97	−1.50	2.30	−13.00, +7.75	−2.15	−1.75	2.42	−13.25, +8.25	−2.30	−2.00	2.51	−13.25, +8.25
2008	−2.07	−1.75	2.26	−12.50, +8.25	−2.30	−2.00	2.43	−10.75, +7.75	−2.45	−2.25	2.47	−12.50, +8.25
2009	−2.25	−1.75	2.35	−11.00, +9.50	−2.46	−2.25	2.43	−12.50, +6.75	−2.59	−2.25	2.49	−12.50, +9.50
2010	−2.34	−2.00	2.43	−11.75, +10.25	−2.53	−2.25	2.54	−11.75, +8.25	−2.68	−2.50	2.60	−11.75, +10.25
2011	−2.42	−2.00	2.46	−12.00, +7.25	−2.62	−2.25	2.57	−12.75, +9.75	−2.76	−2.50	2.64	−12.75, +9.75
2012	−2.47	−2.00	2.45	−11.25, +8.75	−2.70	−2.50	2.58	−11.50, +10.75	−2.86	−2.50	2.63	−11.50, +10.75
2013	−2.54	−2.25	2.50	−12.50, +7.75	−2.78	−2.50	2.60	−13.00, +7.25	−2.93	−2.75	2.66	−13.00, +7.75
2014	−2.68	−2.50	2.46	−13.00, +9.25	−2.89	−2.75	2.55	−13.25, +8.25	−3.07	−2.75	2.61	−13.25, +9.25
2015	−2.75	−2.50	2.51	−12.75, +8.25	−2.95	−2.75	2.61	−12.75, +8.50	−3.13	−2.75	2.66	−12.75, +8.50
Total	−2.34	−2.00	2.42	−13.00, +10.25	−2.54	−2.25	2.52	−13.00, +10.75	−2.70	−2.50	2.59	−13.25, +10.75

^a^ *SD* = standard deviation; ^b^ Min = minimum; ^c^ Max = Maximum

### Incidence of myopia

**Fig. 1:**
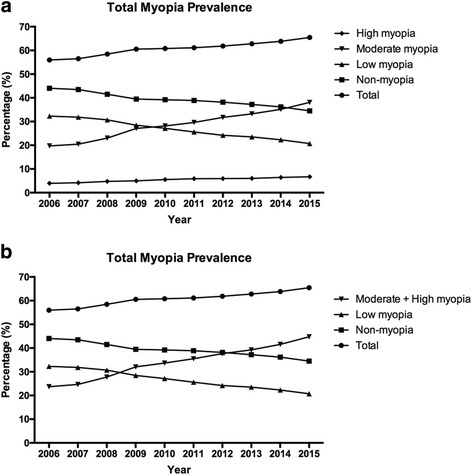
Prevalence of non-myopia and myopia in the whole group. **a** The incidence of non-myopia, low myopia, moderate myopia, and high myopia are presented. The total myopia group was defined using a refractive error > −0.50 diopters and included students with low myopia, moderate myopia, and high myopia. Overall, the prevalence of myopia increased significantly. **b** The total myopia group was defined using a refractive error > −3.0 diopters and included the moderate myopia and high myopia groups

**Fig. 2:**
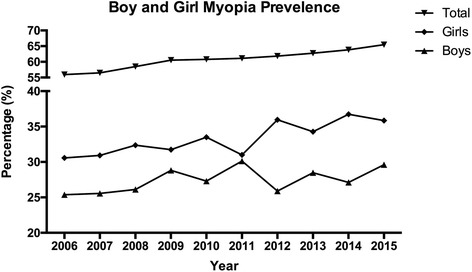
Prevalence of myopia in boys and girls. In a univariate analysis, girls were significantly more myopic and more likely to have myopia than boys from 2006 to 2015

The prevalence of myopia was defined as a refractive error ≤ -0.50 diopters, as described in the Methods section. According to our data, the proportion of students with non-myopia (44.05% in 2006 to 34.52% in 2015) and low myopia (32.27% in 2006 to 20.73% in 2015) decreased during the study period, whereas there was a significant increase in the proportion of students with moderate myopia (19.72% in 2006 to 38.06% in 2015) and high myopia (3.96% in 2006 to 6.69% in 2015). Overall, the prevalence of myopia increased significant from 55.95% in 2006 to 65.48% in 2015 (a 9.53% increase, *P* < 0.001, Fig. 1, Fig. 2, and Table 3). The moderate high myopia subgroup increased by 18.34%, and this was the most significantly increase out of all of the subgroups (Fig. 1b, Table 2). The prevalence of moderate and high myopia exceeded that of low myopia after 2009 and surpassed the prevalence of non-myopia after 2012 (Fig. 1b).

**Table 3:** Prevalence of myopia among third-year students in junior high school in Haidian District, Beijing, China

YearNumber (Percentage, %)	2006 *n* (%)	2007 *n* (%)	2008 *n* (%)	2009 *n* (%)	2010 *n* (%)	2011 *n* (%)	2012 *n* (%)	2013 *n* (%)	2014 *n* (%)	2015 *n* (%)	Total *n* (%)
non-myopia^a^	1611 (44.05)	1573 (43.51)	1521 (41.53)	1459 (39.46)	1528 (39.21)	1471 (38.87)	1456 (38.16)	1410 (37.23)	1386 (36.16)	1269 (34.52)	14,684 (100)
Boys	814 (22.26)	796 (22.02)	772 (21.08)	768 (20.77)	789 (20.25)	786 (20.77)	764 (20.02)	737 (19.46)	744 (19.41)	683 (18.58)	7653 (20.45)
Girls	797 (21.79)	777 (21.49)	749 (20.45)	691 (18.69)	739 (18.96)	685 (18.10)	692 (18.13)	673 (17.77)	642 (16.75)	586 (15.94)	7031 (18.79)
low myopia	1180 (32.27)	1151 (31.84)	1124 (30.69)	1052 (28.46)	1057 (27.12)	969 (25.61)	923 (24.19)	890 (23.50)	854 (22.28)	762 (20.73)	9962 (26.62)
Boys	603 (16.49)	577 (15.96)	571 (15.59)	568 (15.36)	558 (14.32)	539 (14.24)	398 (10.43)	467 (12.33)	461 (12.03)	402 (10.94)	5144 (13.75)
Girls	577 (15.78)	574 (15.88)	553 (15.10)	484 (13.09)	499 (12.80)	430 (11.36)	525 (13.76)	423 (11.17)	393 (10.25)	360 (9.79)	4818 (12.87)
moderate myopia	721 (19.72)	740 (20.47)	843 (23.02)	1002 (27.01)	1097 (28.15)	1121 (29.62)	1211 (31.73)	1259 (33.25)	1347 (35.14)	1399 (38.06)	10,740 (28.70)
Boys	289 (7.90)	311 (8.60)	332 (9.07)	427 (11.55)	420 (10.78)	512 (13.53)	492 (12.89)	528 (13.94)	501 (13.07)	592 (16.10)	4273 (11.42)
Girls	432 (12.81)	429 (11.87)	511 (13.95)	575 (15.55)	677 (17.37)	609 (16.09)	719 (18.84)	731 (19.30)	846 (22.07)	807 (21.95)	6467 (17.28)
high myopia	145 (3.96)	151 (4.18)	174 (4.75)	184 (4.98)	215 (5.52)	223 (5.89)	226 (5.92)	228 (6.02)	246 (6.42)	246 (6.69)	2038 (5.45)
Boys	36 (0.98)	36 (1.00)	53 (1.45)	70 (1.89)	86 (2.21)	89 (2.35)	98 (2.57)	84 (2.22)	77 (2.01)	95 (2.58)	724 (1.93)
Girls	109 (2.98)	115 (3.18)	121 (3.30)	114 (3.08)	129 (3.31)	134 (3.54)	128 (3.35)	144 (3.80)	169 (4.41)	151 (4.11)	1314 (3.51)
Total	3657 (100.00)	3615 (100.00)	3662 (100.00)	3697 (100.00)	3897 (100.00)	3784 (100.00)	3816 (100.00)	3787 (100.00)	3833 (100.00)	3676 (100.00)	37,424 (100)
Boys	1742 (47.63)	1629 (45.06)	1718 (46.91)	1833 (49.58)	1853 (47.55)	1926 (50.90)	1752 (45.91)	1816 (47.95)	1783 (46.52)	1772 (48.20)	17,794 (47.55)
Girls	1915 (52.37)	1986 (54.94)	1944 (53.09)	1864 (50.42)	2044 (52.45)	1858 (49.10)	2064 (54.09)	1971 (52.05)	2050 (53.48)	1904 (51.80)	19,630 (52.45)

^a^non-myopia: emmetropia and hyperopia

### Males versus females

**Fig. 3:**
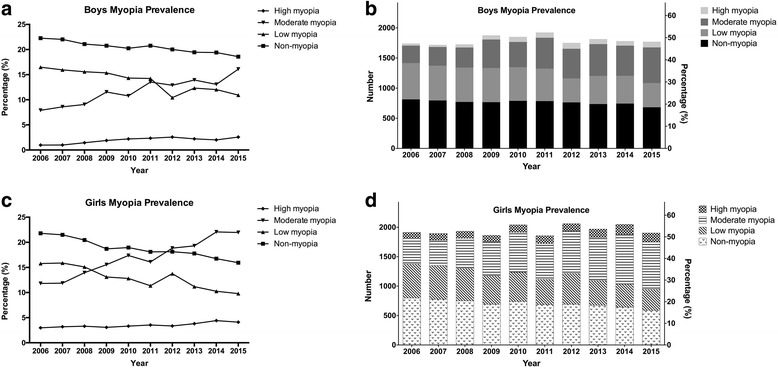
Prevalence of myopia in each myopic group in boys and girls. **a** and **b**, the prevalence of non-myopia, low myopia, moderate myopia, and high myopia in boys. **c** and **d**, the prevalence of non-myopia, low myopia, moderate myopia, and high myopia in girls

In a univariate analysis, girls were significantly (*P* < 0.001) more myopic and more likely to have myopia than boys (Fig. 2, Chi-squared test, *P* < 0.05 for each year except 2011; and Fig. 3). The percentage of students with myopia was already 30.57% in girls in 2006, and this proportion subsequently increased, reaching 35.85% in 2015 (a 5.28% increase, *P* < 0.001). In contrast, the percentage of boys with myopia increased from 25.38% in 2006 to 29.62% in 2015 (a 4.24% increase, *P* < 0.001). In 2015, the prevalence of myopia was 6.23% higher in girls than in boys. Between females and males, the odds ratio (OR, 95% confidence interval (CI)) for overall myopia was 1.43 (95% CI: 1.14–1.96, *P* = 0.007).

**Fig. 4:**
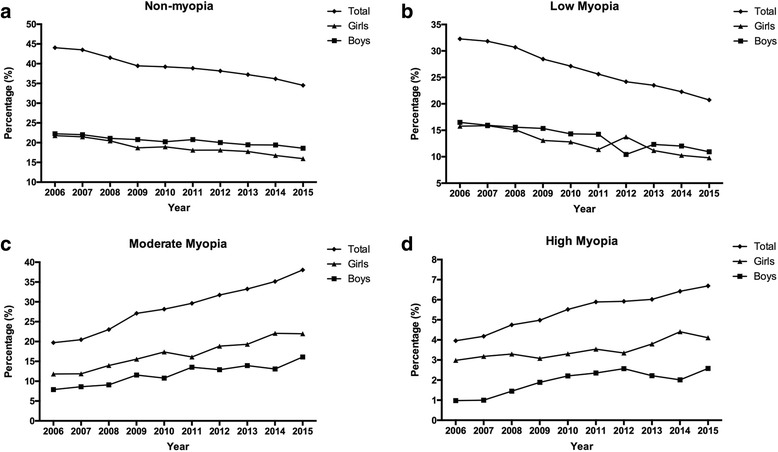
The prevalence of each myopia subgroup in girls and boys. **a**, **b**, **c** and **d** represent non-myopia, low myopia, moderate myopia, and high myopia prevalence separately in boys and girls. The results indicate that females are more likely than males to develop moderate myopia

In both boys and girls, the proportion of students with low myopia decreased significantly between 2006 and 2015 (both *P* < 0.001), and the changes in the proportions between the two subgroups occurred in parallel (Fig. 4). However, in the female subgroup, the prevalence of students with moderate and high myopia increased more than in the male group. In the moderate myopia group, the prevalence in girls increased by 9.14%, from 12.81% in 2006 to 21.95% in 2015 (Fig. 4c), and this was more significant than the increase in the boys group (*P* < 0.001). However, the prevalence of high myopia in girls increased by 0.53%, from 2.98% in 2006 to 3.51% in 2015 (Fig. 4d), but there is no statistically significant change in the boys group, which increased by 0.95%, from 0.98% in 2006 to 1.93% in 2015. In the girls subgroup, the prevalence of moderate myopia surpassed the prevalence of low myopia after 2009, whereas in the boys group, this event occurred after 2012 (Fig. 3). These findings indicate that females are more likely than males to develop moderate myopia (*P* < 0.001).

## Discussion

There are three main findings in the present study, which was performed from 2006 to 2015 in a population of third-year students (aged 15 to 16 years old) in junior high school (grade 9) in the Haidian District of Beijing, China. First, the mean spherical equivalent refractive error and prevalence of myopia increased significantly, according to measurements of cycloplegic autorefractioin between baseline and follow-up examinations. Second, the proportion of students with hyperopia, emmetropia and low myopia decreased over the study period, while the prevalence of myopia increased significantly. Finally, girls were significantly more myopic and more likely to have myopia than boys, especially in the moderate and high myopia subgroups.

Myopia is an important disease that causes visual impairment in individuals around the world, especially children [1, 13]. Correcting an individual’s myopia status is the most cost-effective issue in public eye health care [14, 15]. Moreover, as an often underestimated health condition, myopia is associated with a variety of vision-threatening ocular complications, such as maculopathy, choroidal neovascularization and retinal detachment [16–18].

During recent decades, multiple population-based surveys have been performed in different areas that have reported the prevalence of myopia. For example, the myopia prevalence is 22.3% in Iran [19], 23.7% in Western Australia [20], and 28.3% in Israel [21]. However, the prevalence of myopia is much higher in East Asia, where it has been found to be as high as 84% in Taiwan [3] and 81.6% in Singapore [7]. In China, according to the results of the National Survey on the Constitution and Health of Chinese Students, there has been a remarkable increase in the number of individuals with reduced VA, from 50.8% in 1985 to 79.3% in 2010 [22]. Additionally, other studies have demonstrated that the prevalence of myopia is higher as 95.5% in Shanghai [8], 80% in Shandong [23], and 74.2% in Beijing [24]. The incidence reported in these studies showed a great deal of variation, but the observed trend indicates that East Asians tend to be more likely to experience a myopic shift in refraction, perhaps because of variations in environment, geography, and other factors [25, 26].

Nevertheless, although an abundance of cross-sectional studies have explored the prevalence of myopia in school-aged children, there have been relatively few longitudinal follow-up studies of any specific group. Several studies have reported on the continuous incidence of myopia; for example, in Taiwan, the prevalence of myopia in 18-year-old children increased from 74% in 1983 to 84% in 2004 [3]. Koh et al. conducted a survey in Singapore and found that the overall prevalence of myopia increased in young males from 79.2% in 1996–1997 to 81.6% in 2009–2010 [7]. In mainland China, a longitudinal study of refractive error was performed in Shunyi District in 2000, and it reported that the annualized incidence of myopia was 7.8%, while the rate of myopic progression was −0.17 D per year [27, 28]. In 2016, another population-based cross-sectional survey of refractive error was performed in children aged 6 to 15 years old in Chonqing City, Western China, and a subsequent 5-year longitudinal follow-up study reported an annual progression of refraction in a myopic direction of −0.43 D in this population [12]. However, these studies of prevalence have primarily focused on all school-aged children, including primary school students and students who are up to 18 years old.

As stated in the Introduction, the third year of junior high school (grade 9) and the third year of senior high school (grade 12) are times of particularly strong study pressure. The Guangzhou study showed that there was a significant increase in the prevalence of severely and moderately reduced VA, from 62.5% in 1988 to 84.1% in 2007, in grade 12 students [29]. Another survey from the National Survey on the Constitution and Health of Chinese Students in mainland China also showed that the prevalence of reduced VA in 16- to18-year-old subjects significantly increased from 50.8% in 1985 to 79.3% in 2010 [22]. In the present study, we performed a 10-year longitudinal follow-up study to investigate changes in the prevalence of myopia in the third year of the Haidian District of Beijing, China. This region is associated with the most intense learning stress in Beijing. Similar to a report from the Beijing Childhood Eye Study, we found that the prevalence of myopia in 14 to 16-year-olds was between 61.9% and 73%. Our data also showed similar patterns of changes to those observed in previous studies, in that we observed a remarkable and significant increase in the prevalence of myopia from 55.95% in 2006 to 65.48% in 2015. The moderate high myopia subgroup increased the most significantly among all of the subgroups, with an 18.34% increase.

Our findings that the progression of myopia as also associated with being female and having a higher level of baseline myopia are similar to the findings described in a previous report. Previously, studies in Shunyi [28], Yongchuan [12], Finland [30] and Australia [20] also reported observing an increase in the progression of myopia and a tendency for female individuals to be more or more frequently myopic. In our survey, in which we included both boys and girls, the proportion of students in both low myopia sub-groups significantly decreased between 2006 and 2015, and the two subgroups decreased in parallel. However, there was a significantly higher prevalence of moderate and high myopia in female than in male subjects, who showed an increase from 12.81% in 2006 to 21.95% in 2015, consistent with previous studies [8, 23, 31]. One possible explanation is that girls tend to read and write more and to perform less outdoor exercise [32, 33]. The associated increase in near-eye work predisposes them to developing myopia [32]. The school system is becoming more and more competitive in mainland China, especially in terms of the expectation that students must achieve high scores to enter a high quality senior high school. It has been reported that 12.5% of students do not participate in any kind of outdoor activity [33, 34]. A recent study from Guangzhou also reported the prevalence of myopia in students increased along with the growth of grade level, which the prevalence of myopia in students in grade 3 was 38.8%, and the rate was increased to 68.4% in grade 9 [29]. And the prevalence in grade 9 was also consistent with the findings from our study 65.48%. Previous studies showed that a sort duration of outdoor activities is among a number of factors, such as parental myopia and region of habitation, that have major associations with the prevalence and incidence of myopia in children [35]. In comparisons between the sexes, girls usually spend more time reading and performing near-eye work and with less time engaging in outdoor activities, and this makes them more vulnerable to developing myopia [24].

Several methodological limitations should be acknowledged. For example, in the present study a questionnaire was not used to analyze the presence of possible risk factors for myopia, such as parental myopia, time spent in outdoor activities, and ethnicity. Additionally, we did not measure axial length, which would have complemented our data. Finally, strict statistical randomization was not applied in our survey. Further prospective, large-scale, multi-center studies are therefore required to validate our data.

To our knowledge, this is the first longitudinal study to examine the incidence and progression of myopia in third year students (grade 9, ages 15 to 16 years old) in junior high school in the Haidian District of Beijing, China. During the 10-year study period, from 2006 to 2015, the mean spherical equivalent refractive error and prevalence of myopia significantly increased, and the incidence of myopia also significantly increased. And according to our data, girls were significantly more myopic and more likely to have myopia than boys, especially in the moderate myopia subgroup.

## Conclusions

In the present study, we investigated the prevalence of myopia from 2006 to 2015 in a population of third-year students in junior high school in the Haidian District of Beijing, China. During the past 10 years, the prevalence of myopia significantly increased on an annual basis, especially the moderate and high myopia population. Meanwhile, girls were significantly more myopic and more likely to have myopia than boys. The refractive status and accordingly performed intervention strategies deserves particular attention for the whole society of China.

## Additional file

Additional file 1: The file attached includes the raw data of the refractive status of all individual in the present study, which from 2006 to 2015 in the junior high school in the Haidian District of Beijing, China. In each tab page, the patent ID of the students were recorded by number; the gender was recorded by male (M = 1) or female (F = 2); the refractive status of the right eye (OD), left eye (OS), and the worse eye were also recorded by diopter. At the end of each tab page, the mean, standard deviation (SD), and median of the refractive status of all individual in each year were also calculated. (XLSX 2605 kb)

## Abbreviations

BCVA: Best-corrected visual acuity
CI: Confidence intervals
D: Diopters
IQR: Interquartile range
ORs: Odds ratios
RE: Refractive error
SER: Spherical equivalent refraction
SERE: Spherical equivalent refractive error
VA: Visual acuity

## References

[1] Morgan IG, Ohno-Matsui K, Saw SM (2012). Myopia. Lancet.

[2] Pan CW, Ramamurthy D, Saw SM (2012). Worldwide prevalence and risk factors for myopia. Ophthalmic Physiol Opt.

[3] Lin LL, Shih YF, Hsiao CK, Chen CJ (2004). Prevalence of myopia in Taiwanese schoolchildren: 1983 to 2000. Ann Acad Med Singap.

[4] Matsumura H, Hirai H (1999). Prevalence of myopia and refractive changes in students from 3 to 17 years of age. Surv Ophthalmol.

[5] Jung SK, Lee JH, Kakizaki H, Jee D (2012). Prevalence of myopia and its association with body stature and educational level in 19-year-old male conscripts in seoul, South Korea. Invest Ophthalmol Vis Sci.

[6] Lee JH, Jee D, Kwon JW, Lee WK (2013). Prevalence and risk factors for myopia in a rural Korean population. Invest Ophthalmol Vis Sci.

[7] Koh V, Yang A, Saw SM, Chan YH, Lin ST, Tan MM, et al. Differences in prevalence of refractive errors in young Asian males in Singapore between 1996-1997 and 2009-2010. Ophthalmic Epidemiol. 2014;21(4):247–55. 10.3109/09286586.2014.928824 24990474

[8] Sun J, Zhou J, Zhao P, Lian J, Zhu H, Zhou Y, et al. High prevalence of myopia and high myopia in 5060 Chinese university students in shanghai. Invest Ophthalmol Vis Sci. 2012;53(12):7504–9. 10.1167/iovs.11-8343 23060137

[9] Wu LJ, You QS, Duan JL, Luo YX, Liu LJ, Li X, et al. Prevalence and associated factors of myopia in high-school students in Beijing. PLoS One. 2015;10(3):e0120764. 10.1371/journal.pone.0120764 PMC4372519 25803875

[10] Kang MT, Li SM, Peng X, Li L, Ran A, Meng B, et al. Chinese eye exercises and myopia development in school age children: a nested case-control study. Sci Rep. 2016;6:28531. 10.1038/srep28531 PMC4916489 27329615

[11] Yu S, Diao H, Zeng J (2015). Analysis of the prevalence and situation of myopia in adolescents from South China. Eye Sci.

[12] Zhou WJ, Zhang YY, Li H, Wu YF, Xu J, Lv S, et al. Five-year progression of refractive errors and incidence of myopia in school-aged children in Western China. J Epidemiol. 2016;26(7):386–95. 10.2188/jea.JE20140258 PMC4919484 26875599

[13] Rudnicka AR, Kapetanakis VV, Wathern AK, Logan NS, Gilmartin B, Whincup PH, et al. Global variations and time trends in the prevalence of childhood myopia, a systematic review and quantitative meta-analysis: implications for aetiology and early prevention. Br J Ophthalmol. 2016; 10.1136/bjophthalmol-2015-307724 PMC4941141 26802174

[14] Choo V (2003). A look at slowing progression of myopia. Lancet.

[15] Repka MX (2015). Prevention of myopia in children. JAMA.

[16] Wong TY, Ferreira A, Hughes R, Carter G, Mitchell P (2014). Epidemiology and disease burden of pathologic myopia and myopic choroidal neovascularization: an evidence-based systematic review. Am J Ophthalmol.

[17] Xu L, Wang Y, Li Y, Wang Y, Cui T, Li J, et al. Causes of blindness and visual impairment in urban and rural areas in Beijing: the Beijing eye study. Ophthalmology. 2006;113(7):1134 e1131–11. 10.1016/j.ophtha.2006.01.035 16647133

[18] Ohno-Matsui K, Yoshida T (2004). Myopic choroidal neovascularization: natural course and treatment. Curr Opin Ophthalmol.

[19] Hashemi H, Rezvan F, Ostadimoghaddam H, Abdollahi M, Hashemi M, Khabazkhoob M (2013). High prevalence of refractive errors in a rural population: 'Nooravaran Salamat' mobile eye Clinic experience. Clin Experiment Ophthalmol.

[20] McKnight CM, Sherwin JC, Yazar S, Forward H, Tan AX, Hewitt AW, et al. Myopia in young adults is inversely related to an objective marker of ocular sun exposure: the Western Australian Raine cohort study. Am J Ophthalmol. 2014;158(5):1079–85. 10.1016/j.ajo.2014.07.033 PMC4786165 25072831

[21] Bar Dayan Y, Levin A, Morad Y, Grotto I, Ben-David R, Goldberg A, et al. The changing prevalence of myopia in young adults: a 13-year series of population-based prevalence surveys. Invest Ophthalmol Vis Sci. 2005;46(8):2760–5. 10.1167/iovs.04-0260 16043848

[22] Sun HP, Li A, Xu Y, Pan CW (2015). Secular trends of reduced visual acuity from 1985 to 2010 and disease burden projection for 2020 and 2030 among primary and secondary school students in China. JAMA Ophthalmol.

[23] Wu JF, Bi HS, Wang SM, Hu YY, Wu H, Sun W, et al. Refractive error, visual acuity and causes of vision loss in children in Shandong, China. The Shandong children eye study. PLoS One. 2013;8(12):e82763. 10.1371/journal.pone.0082763 PMC3871613 24376575

[24] You QS, Wu LJ, Duan JL, Luo YX, Liu LJ, Li X, et al. Prevalence of myopia in school children in greater Beijing: the Beijing childhood eye study. Acta Ophthalmol. 2014;92(5):e398–406. 10.1111/aos.12299 25165786

[25] Aller TA (2014). Clinical management of progressive myopia. Eye (Lond).

[26] Ramamurthy D, Lin Chua SY, Saw SM (2015). A review of environmental risk factors for myopia during early life, childhood and adolescence. Clin Exp Optom.

[27] Zhao J, Pan X, Sui R, Munoz SR, Sperduto RD, Ellwein LB (2000). Refractive error study in children: results from Shunyi District, China. Am J Ophthalmol.

[28] Zhao J, Mao J, Luo R, Li F, Munoz SR, Ellwein LB (2002). The progression of refractive error in school-age children: Shunyi district, China. Am J Ophthalmol.

[29] Guo L, Yang J, Mai J, Du X, Guo Y, Li P, et al. Prevalence and associated factors of myopia among primary and middle school-aged students: a school-based study in Guangzhou. Eye (Lond). 2016;30(6):796–804. 10.1038/eye.2016.39 PMC4906452 26965016

[30] Parssinen O, Kauppinen M, Viljanen A (2014). The progression of myopia from its onset at age 8-12 to adulthood and the influence of heredity and external factors on myopic progression. A 23-year follow-up study. Acta Ophthalmol.

[31] Guo YH, Lin HY, Lin LL, Cheng CY (2012). Self-reported myopia in Taiwan: 2005 Taiwan National Health Interview Survey. Eye (Lond).

[32] Saw SM, Zhang MZ, Hong RZ, Fu ZF, Pang MH, Tan DT (2002). Near-work activity, night-lights, and myopia in the Singapore-China study. Arch Ophthalmol.

[33] He M, Xiang F, Zeng Y, Mai J, Chen Q, Zhang J, et al. Effect of time spent outdoors at school on the development of myopia among children in China: a randomized clinical trial. JAMA. 2015;314(11):1142–8. 10.1001/jama.2015.10803 26372583

[34] Rose KA, Morgan IG, Ip J, Kifley A, Huynh S, Smith W, et al. Outdoor activity reduces the prevalence of myopia in children. Ophthalmology. 2008;115(8):1279–85. 10.1016/j.ophtha.2007.12.019 18294691

[35] Wu PC, Tsai CL, Wu HL, Yang YH, Kuo HK (2013). Outdoor activity during class recess reduces myopia onset and progression in school children. Ophthalmology.

